# Treatment Strategies for Glioblastoma in the Elderly: What Should We Focus on Compared to Younger Patients

**DOI:** 10.3390/cancers16061231

**Published:** 2024-03-21

**Authors:** Hanah Hadice Gull, Antonia Carlotta Von Riegen, Greta Theresa Beckmann, Pikria Ketelauri, Sebastian Walbrodt, Alejandro N. Santos, Christoph Oster, Teresa Schmidt, Martin Glas, Ramazan Jabbarli, Neriman Özkan, Philipp Dammann, Björn Scheffler, Ulrich Sure, Yahya Ahmadipour

**Affiliations:** 1 Department of Neurosurgery and Spine Surgery, University Hospital Essen, University of Duisburg-Essen, 45147 Essen, Germany; antoniacarlotta.vonriegen@uk-essen.de (A.C.V.R.); greta.beckmann@gmx.de (G.T.B.); sebastian.walbrodt@uk-essen.de (S.W.); alejandro.santos@uk-essen.de (A.N.S.); philipp.dammann@uk-essen.de (P.D.);; 2 Center for Translational Neuro- and Behavioral Sciences (C-TNBS), University of Duisburg-Essen, 45147 Essen, Germany; martin.glas@uk-essen.de; 3 DKFZ-Division Translational Neurooncology at the WTZ, DKTK Partner Site, University Hospital Essen, 45147 Essen, Germany; christoph.oster@uk-essen.de (C.O.); teresa.schmidt@uk-essen.de (T.S.);; 4 West German Cancer Center (WTZ), University Hospital Essen, 45147 Essen, Germany; 5 Department of Neurology, Center for Translational Neuro- and Behavioral Sciences (C-TNBS), Division of Clinical Neurooncology, University Medicine Essen, University of Duisburg-Essen, 45147 Essen, Germany; 6 German Cancer Consortium (DKTK), 69120 Heidelberg, Germany; 7 German Cancer Research Center (DKFZ), 69120 Heidelberg, Germany; 8 Center of Medical Biotechnology (ZMB), University of Duisburg-Essen, 45147 Essen, Germany

**Keywords:** glioblastoma, elderly patients, progression-free survival, overall survival, elderly glioblastoma patients

## Abstract

**Simple Summary:**

Although the incidence of glioblastoma (GB) in people over 65 years is more than twice as high as in younger people, no standard of care for the treatment of the elderly with GB has been established so far. With this study, we aimed to investigate the predictors affecting outcomes in elderly GB patients with regard to their overall survival (OS) and progression-free survival (PFS). MGMT promoter methylation, gross total resection (GTR), and hypofractionated radiation were associated with a longer OS in elderly glioblastoma patients. These findings may induce the optimization of the treatment regimen for elderly patients that has not yet been established.

**Abstract:**

(1) Background: Although the incidence of glioblastoma (GB) has a peak in patients aged 75–84 years, no standard treatment regimen for elderly patients has been established so far. The goal of this study was to analyze the outcome of GB patients ≥ 65 years to detect predictors with relevant impacts on overall survival (OS) and progression-free survival (PFS). (2) Methods: Medical records referred to our institution from 2006 to 2020 were analyzed. Adult GB patients with clinical data, postoperative MRI data, and ≥1 follow-up investigation after surgical resection were included. The complete cohort was divided into a younger (<65) and an elderly group (≥65 years). Multiple factors regarding OS and PFS were scanned using univariate and multivariable regression with *p* < 0.05. (3) Results: 1004 patients were included with 322 (61.0%) male individuals in the younger and 267 (56.1%) males in the older cohort. The most common tumor localization was frontal in both groups. Gross total resection (GTR) was the most common surgical procedure in both groups, followed by subtotal resection (STR) (145; 27.5%) in the younger group, and biopsy (156; 32.8%) in the elderly group. Multivariate analyses detected that in the younger cohort, MGMT promoter methylation and GTR were predictors for a longer OS, while MGMT methylation, GTR, and hypofractionated radiation were significantly associated with a longer OS in the elderly group. (4) Conclusions: Elderly patients benefit from surgical resection of GB when they show MGMT promoter methylation, undergo GTR, and receive hypofractionated radiation. Furthermore, MGMT methylation seems to be associated with a longer PFS in elderly patients. Further investigations are required to confirm these findings, especially within prospective radiation therapy studies and molecular examinations.

## 1. Introduction

Despite multimodal treatment including microsurgical tumor resection followed by combined radio (RT)- and temozolomide (TMZ)-based chemotherapy, glioblastoma patients develop disease progression. As a result, the median survival after standard-of-care treatment is about ~18 months with survival after progression at 6–8 months [1]. Several predictors regarding the prognosis of glioma, such as initial clinical condition, age of the patient, extent of resection, molecular characteristics like mutation of the isocitrate-dehydrogenase 1 (IDH1) gene, and methylation of the O6-methylguanin-DNA-methyltransferase (MGMT) promotor are already described [2,3,4,5,6], but their significance regarding different age groups is not sufficiently investigated [2,7,8]. In particular, no clear indication exists for elderly glioblastoma patients ≥ 65 years, as no standard of care for the treatment of the elderly with glioblastoma (GB) has been established so far [9,10,11], although the incidence of GB in people over 65 years is more than twice as high as in younger people [12], it has a peak in patients aged from 75–84 years, and the population will be constantly increasing [13]. The key treatment recommendations at diagnosis for adult patients with common diffuse gliomas according to the EANO guidelines consist of temozolomide chemoradiotherapy (54–60 Gy in 1.8–2 Gy fractions); for patients aged > 65–70 years and with MGMT unmethylated tumors, radiotherapy (40 Gy in 2.67 Gy fractions); for patients aged > 65–70 years and with MGMT methylated tumors, temozolomide chemoradiotherapy or temozolomide only [14]. Treatment recommendations for progression or recurrence for these patients are nitrosourea, temozolomide rechallenge, bevacizumab, and radiotherapy (for patients not previously treated with radiotherapy) [15,16,17,18,19]. However, elderly glioblastoma patients are less likely to receive combined radiation and chemotherapy after surgical resection [20,21] resulting in a worse prognosis [22]. In addition, Pretanvil et al. determined that the therapeutic effect of elderly GB patients gradually deteriorates with age; however, surgical debulking followed by radiation and chemotherapy leads to an improved OS [20]. Using a large institutional cohort in the present study, we aimed to investigate the course of the disease glioblastoma in elderly patients. Furthermore, using this large cohort, we aimed to detect the predictors affecting outcomes in elderly GB patients. This may provide doctors with comprehensive information achieving a better understanding and treatment of this disease in the elderly population. 

## 2. Materials and Methods

### 2.1. Data Collection and Clinical Management

This study was based on an institutional observational database and retrospectively included newly diagnosed glioblastoma treated between January 2006 and December 2020 in our neurosurgical department. It was conducted according to the STROBE guidelines. 

Histological evaluation according to the 2021 Classification of the Central Nervous System Tumors of the World Health Organization (WHO) approved the diagnosis after stereotactic biopsy or tumor resection [23]. IDH1/2 mutated GB were completely excluded to conform to the recent WHO classification ensuring consistency in diagnosis and reduction in patient selection bias [23]. After tumor resection, an early postoperative MRI within 72 h was performed to evaluate the extent of resection, categorized as gross total resection by the absence of an enhancing lesion on T1-weighted contrast-enhanced images and as debulking in remaining instances. If the patient’s neurological and clinical status were appropriate as measured by the Karnofsky performance status scale (KPS), standard chemoradiation with simultaneous and adjuvant temozolomide was performed after surgery. Repeated follow-ups with MRI, and if required with positron emission tomography (PET) imaging at various time intervals, such as 2–3 months or earlier in the case of clinical deterioration were used to distinguish true progression from pseudoprogression. Demographic data (age and sex), anthropometric parameters (body height, weight, and body mass index), clinical characteristics (KPS at admission and discharge, medical history), tumor characteristics (location, immunohistochemical and molecular genetic parameters [24], such as expression of Ki-67 proliferation index, isocitrate dehydrogenase 1 gene (IDH) mutation [7], O6- methylguanine DNA methyltransferase [10] promoter methylation status), and extent of resection (EOR; gross total resection (GTR, >95% by volume) vs. subtotal resection (STR, ≤95%) vs. biopsy at initial consultation (stereotactic and open)), and outcome (progression-free survival, overall survival or date of the last outpatient follow-up) were analyzed. Pediatric patients (<18 years old), patients with an extracranial location, and incomplete data sets were excluded. OS was defined as the period from first diagnosis to the date of glioblastoma-related death. The appearance of tumor progression (PFS) was defined according to the recent RANO (Response Assessment in Neuro-Oncology) criteria [25].

### 2.2. Cohort Division

We categorized the entire cohort into two groups as follows: patients with diagnosed glioblastoma < 65 and ≥ 65 years old. Based on leading international studies like the Stupp trial selecting cutoff values of 70 years [15] and 60 years [22], or the Nordic trial including patients > 60 years [19], we have decided to choose the golden mean and take 65 years as the cutoff [8,26,27]. According to the 2021 Classification of the Central Nervous System Tumors of the WHO, we retrospectively excluded all patients with diffuse glioma and IDH mutation [23]. 

### 2.3. Study Endpoints and Statistical Analysis

The effect of different parameters on OS and PFS were the study endpoints in both groups, measured from the date of diagnosis to death from glioblastoma. The different parameters could be divided into different sections, such as tumor data (localization, molecular status), patient data (sex, age, Karnofsky Performance Index, Charlson Comorbidity Index), surgical data (extent of resection), and postoperative data (radiation, relapse, overall survival). For all statistical analyses, we used SPSS 26 (IBM Corporation, Armonk, New York, NY, USA). Univariate analyses were performed to determine predictors of poor or good outcomes at the first and last follow-ups. For dichotomized variables, the χ2 test was used. Continuous variables were analyzed using the Student’s *t*-test (normally distributed data) or the Mann–Whitney U test (non-normally distributed data). Kendall’s tau-b was assessed for continuous and ordinal variables; Spearman’s rho was used for continuous and dichotomous variables. Predictive factors regarding outcomes were assessed by the calculation of odds ratios (ORs) and 95% confidence intervals (95% Cis) using logistic, linear, or ordinal regression models. Significant parameters detected through univariate analysis as well as parameters with *p* values  <  0.1 were ultimately assessed using multivariate analysis. 

### 2.4. Ethics

This study was conducted in accordance with the Strengthening the Reporting of Observational Studies in Epidemiology (STROBE) guidelines after approval by the Institutional Review Board (Medical Faculty, University of Duisburg-Essen, Registration Number: 15-6504-BO) and followed The Code of Ethics of the World Medical Association (Declaration of Helsinki).

## 3. Results

### 3.1. Patient Demographics

A total of 1004 patients were included in this study. The mean age in the complete cohort was 61.91 ± 13.38 years with a range from 19.3 to 88.1 years and with 415 (41.6%) female and 589 (58.3%) male individuals. Furthermore, 476 of 1004 patients diagnosed with glioblastoma from 2006 to 2020 were ≥65 years. The most frequent tumor localization was in the frontal lobe in both age groups. Concerning clinical presentation, a seizure as the first symptom was observed in 185 (35.0%) cases in the younger group and in 121 (25.4%) cases in the elderly group. The extent of resection in patients older than 65 years was mainly gross total resection (GTR) in 229 (48.1%) patients, followed by biopsy in 156 (32.8%) patients, and then debulking in 91 (19.1%) patients. In contrast, in the younger group, debulking was the second most common extent of resection (145, 27.5%), directly after GTR in 267 (50.6%) patients. Biopsy was conducted in 116 (22.0%) cases. The mean KPS at admission in patients < 65 years was 83.34 ± 13.52, and 78.84 ± 14.9 in patients ≥ 65 years. At discharge, the mean KPS was 78.7 ± 16.32 in the younger group, and 70.54 ± 20.49 in the elderly group. Regarding postoperative therapy with radiation, we determined that the mean total/cumulative dose was 58.3 ± 6.3 Gy in the younger group, while the elderly group received a mean total dose of 51.6 ± 14.5 Gy. Moreover, we investigated the Ki-67 proliferation index. Here, the mean Ki-67 proliferation index was 18.96 ± 14.16% in glioblastoma patients < 65 years old and 16.04 ± 10.70% in glioblastoma patients ≥ 65 years old. Detailed baseline population information is shown in Table 1.

Furthermore, we performed subgroup analyses concerning OS and PFS; In the younger group, female patients had a longer OS than the male patients (16.77 ± 15.88 months vs. 14.73 ± 17.97) while female and male patients had approximately the same PFS length (11.94 ± 11.38 and 12.90 ± 15.76 months). At the same time, in elderly GB patients, male patients had a longer OS and PFS (10.87 ± 14.23 vs. 8.95 ± 13.13 and 10.51 ± 18.63 and 9.88 ± 13.24 months). In both groups, GTR led to the longest OS and PFS compared to SR or biopsy. MGMT methylation was associated with a longer OS and PFS in both groups, especially with a longer PFS in elderly GB patients. Extensive data are listed in Table 2. 

### 3.2. Basic Differences between Glioblastoma Patients < 65 Years and ≥65 Years

Both groups presented no differences regarding sex (*p* = 0.125), tumor localization (*p* = 0.445), GTR as the extent of resection (*p* = 0.445), and differences in comorbidities (*p* = 0.116) (Table 3). The median CCI in the younger group was 2.77, while the median CCI was 3.23 in the elderly group. Mann–Whitney U and Chi-square tests identified significant differences in KPS at admission and at discharge between the groups (*p* < 0.001) (Table 3). The younger group showed a better KPS at admission as well as at discharge (83.34 ± 13.52 vs. 78.84 ± 14.9 and 78.7 ± 16.32 vs. 70.54 ± 20.49). Furthermore, Mann–Whitney U and Chi-square determined significant differences in radiation dose (*p* < 0.001), MGMT methylation status (*p* = 0.001), in subtotal resection as the extent of resection (*p* = 0.002), and in biopsy (*p* < 0.001), as well as in Ki-67 values (*p* = 0.005) (Table 3). The elderly group was exposed to a lower total radiation dose, presented fewer cases of methylated MGMT status, and underwent biopsy more often (*n* = 156 vs. *n* = 116) after gross total resection (GTR), while the younger group experienced more frequent subtotal resection (*n* = 145 vs. *n* = 91) after GTR and had higher Ki-67 values (18.96 ± 14.16 vs. 16.04 ± 10.70). Detailed characteristics are summarized in Table 1, Table 2 and Table 3.

### 3.3. Basic Predictors Regarding Overall Survival and Progression-Free Survival

Univariate ordinal regression analysis revealed in both groups that MGMT methylation and GTR were statistically significant predictors for a longer OS, while biopsy was significantly associated with a shorter OS in both groups. Furthermore, in the elderly group, a total radiation dose of 51.6 ± 14.5 Gy was correlated significantly with a longer OS. In multivariate analysis, including significant parameters detected through univariate analysis, MGMT methylation and GTR as the extent of resection were confirmed as significant predictors for a longer OS in the younger group, while MGMT methylation, a specific, abovementioned total radiation dose, and GTR were confirmed as significant predictors for a longer OS in the elderly group. At the same time, multivariate analysis detected biopsy as a predictor for a lower OS in the elderly group. Considering the total cohort, a higher age was associated with a lower OS [OR: 0.68, 95% CI: 0.57–0.82, *p* = 0.01]. Regarding progression-free survival, ordinal regression analysis could only detect MGMT methylation as a significant predictor for a longer PFS in the elderly group. Detailed characteristics are listed in Table 4 and Table 5. 

## 4. Discussion

To date, the treatment and management options of elderly patients with glioblastoma have not been thoroughly highlighted [9,21]. In particular, for older patients with glioblastoma, there is currently no consensus on the most appropriate surgery and subsequent adjuvant therapy [21]. Furthermore, the available studies are restricted by small cohort sizes [20]. As the aging population expands, the median age of glioblastoma, which is 64 years, is expected to further increase [28,29]. Therefore, we see the necessity of the examination of elderly GB patients intending to optimize treatment regimens for elderly patients that have not yet been established [20]. Our study focused on the course of glioblastoma in two different cohort groups, younger (<65 years) and elderly (≥65 years) patients, by investigating a set of parameters for OS and PFS. 

### 4.1. OS, PFS, MGMT Methylation, and Ki-67 Proliferation Index

Interestingly, significant differences were neither observed in OS nor in PFS in these two different patient groups in our cohort. This could be explained by the high percentage of elderly patients with MGMT promoter methylation in our cohort that is in accordance with the findings of Connon et al. [28]. In our cohort, methylated MGMT status was observed more frequently in the elderly group than in the younger patients with GB (215 (45.1%) vs. 203 (38.4%), *p* = 0.001). Indeed, MGMT promoter methylation is associated with a longer OS [19] and is a strong predictor of benefit with temozolomide chemotherapy in elderly patients [4,5,17,19,30]. This may explain why OS and PFS are equally “good”. Regarding PFS, our study could not determine significant predictors in the younger cohort. Even MGMT promoter methylation was no significant parameter for a longer PFS. These findings confirm the results of Wang et al. [31]. Considering the elderly group, a trend toward better OS was seen when the lesion was located in the frontal and temporal lobe. We could observe a slight trend toward a better PFS when GTR was conducted as the extent of resection. However, these results did not show statistical significance (Table 4 and Table 5). Contrastingly, our study could also detect a significant predictor for a longer PFS in the elderly group, namely MGMT promoter methylation. This could provide important information about the necessity of further investigation into novel molecular biomarkers in elderly glioblastoma patients [31]. With our study, we could confirm Pretanvil et al.’s findings, namely that in the elderly, the incidence of MGMT methylation is comparable to that of the younger glioblastoma patients. However, Pretanvil et al. reported, that IDH-1 mutations are always absent in the elderly population, which may be one of the reasons for the poorer outcome of elderly patients [20]. According to the 2021 Classification of the Central Nervous System Tumors of the World Health Organization, all of the included patients in our study presented with IDH-1 wild type. Therefore, in this cohort, we cannot distinguish between the impact of IDH-1 mutation or wild type on different glioblastoma age groups. Furthermore, in our study, the most frequent extent of resection in the younger as well as in the elderly group was gross total resection, while in the cohort of Pretanvil et al., elderly glioblastoma patients were more likely to undergo biopsy and less likely to receive combined radiation and chemotherapy [20].

Wang et al. confirmed that elderly patients differ from younger patients in genome, molecular subtypes, epigenetics, and prognostic-related molecular markers [31,32]. Interestingly, in our study, MGMT promoter methylation was associated with a longer PFS not in the younger group, but in the elderly group of glioblastoma patients. 

Furthermore, Wang et al. found out that protein phosphatase 1D (PPM1D), which is a potential prognostic biomarker and correlates with immune cell infiltration in hepatocellular carcinoma [33], was an effective prognostic marker for elderly patients [31] and its silencing was associated with tumor sensitivity to treatment in gliomas [34], while Khadka et al. examined that PPM1D is a good prognostic marker for diffuse midline gliomas [35]. Moreover, Akamandisa et al. found out that PPM1D inhibitors can enhance the anti-proliferative and pro-apoptotic effects of ionizing radiation in diffuse intrinsic pontine glioma [36]. 

Apart from this, Wang et al. observed a correlation between Ki-67 and the age of glioblastoma patients; elderly patients had higher Ki-67 indices compared to younger patients [31]. Furthermore, Liu et al. observed in univariate analysis that higher Ki-67 values (median Ki-67 index > 25%) were associated with worse OS [37]. Interestingly, in our cohort, the elderly glioblastoma patients revealed lower mean Ki-67 values [16.04 ± 10.70% vs. 18.96 ± 14.16%]. This may explain the average OS in the elderly group, which did not significantly differ from the OS in the younger group [13.86 ± 15.73 vs. 15.73 ± 14.93 months, *p* = 0.068]. Additionally, Dahlrot et al. investigated that median Ki-67 values increased with increasing WHO grade; however, median Ki-67 values were not associated with survival in glioblastoma [38]. We could confirm these findings in our study; concerning the complete cohort, higher Ki-67 values were not significantly associated with worse OS [OR: 0.21; 95% CI: 0.12–1.31; *p* = 0155]. Overall, Ki-67 proliferation indices seem to be valuable in some settings, e.g., differential diagnostic settings; however, they should not be over-interpreted in the clinical context [39].

Unfortunately, in this study, we did not investigate PPM1D values in particular with correlation to age. However, we explored a correlation between MGMT promoter methylation and a longer PFS in glioblastoma patients ≥ 65 years emphasizing the importance of molecular and genetic characteristics. So, our study calls for further exploration of the molecular characteristics of elderly patients with glioblastoma intending to determine clear treatment regimes for this subgroup. PPM1D seems to be a valuable prognostic signature molecule, especially in elderly GB patients. Focusing future research directions on the PPM1D status of the tumor and its effect on response to radiation is recommended as part of prospective studies [36].

### 4.2. Radiation in Elderly Glioblastoma Patients

With increasing age, the benefit of chemotherapy decreases, and the risk of cognitive side effects of cranial irradiation increases [11]. So, the effect of radiotherapy on elderly glioblastoma patients should be thoroughly reflected. In our cohort, the radiation total dose in the elderly group differed significantly from the total radiation dose in the younger cohort as it was lower in total (51.6 ± 14.5 Gy vs. 58.3 ± 6.3 Gy in the younger group, *p* < 0.001 in Mann–Whitney U Test) and was a predictor for a longer OS. This confirms the EANO guidelines on the diagnosis and treatment of diffuse gliomas in adulthood [14]. Here, Weller et al. verified that hypofractionated radiotherapy with a higher dose per fraction and a lower total dose is suitable for elderly (>65 to 70 years) glioblastoma patients [14,40]. Furthermore, Perry et al. advised that additional temozolomide to hypofractionated radiotherapy improved OS in patients older than ≥60 years [16]. In our cohort, we did not implement subgroup analysis regarding additional temozolomide during the observation period of our study, as the therapeutical scheme for chemotherapy especially in elderly patients with glioblastoma was constantly changing according to the recent WHO classification [23]. So, we excluded this subgroup analysis to avoid inconsistency and bias in the evaluation. However, hypofractionated radiation seemed to be a predictor of longer OS [41].

In addition, Weller et al. investigated that radiation fractionation depends on tumor size and that larger lesions require smaller single fraction sizes to improve safety and tolerability [14]. Indeed, examinations of tumor size and outcome would be interesting, and more particularly the tumor size and the applied radiation in different age groups of patients with glioblastoma. Measurements of the size of the lesions in our cohorts are missing, but would certainly be relevant for the detection of differences between younger and older glioblastoma patients and the potential implications associated with them [42,43].

### 4.3. Extent of Resection

In both groups, GTR seems to be the matter of choice for a longer OS. Following the literature, our large study approved this method as a standard for the extent of resection as far as surgically and clinically possible [8]. Here, we emphasize the preoperative mean value of the KPI that was >70% even in both groups. Indeed, this KPI is associated with a good prognosis in general [3] and especially in combination with GTR [3,14,44]. Interestingly, despite the older age, the elderly group in our cohort proved to be fit overall as their median CCI was 3.23 and their preoperative KPI was 78.84 ± 14.9%. Moreover, regression analysis revealed no significant differences in KPI and CCI between the elderly and the younger group in our cohort. This may explain why GTR was the most common extent of resection in both groups as GTR is favored when the KPI is ≥70 [15,22]. These results underline the fact that the safest surgical approach was investigated interdisciplinarily to avoid postoperative physical damage. So, when KPI is ≥70, it seems that GTR should be favored regardless of increased age [22].

### 4.4. Limitations

The retrospective study design could be associated with inherent bias and bears the risk of incomplete data and selection biases. Moreover, further parameters could have been investigated. For instance, the interesting aspects not considered in this study were radiological features of the tumor lesion, such as detailed information about tumor size, the necrosis area, concomitant edema, or subventricular zone involvement in the different cohorts [45].

In addition, broad data about molecular and genetic characteristics or extensive data on postoperative systemic therapy in different age groups were potential parameters that could have been investigated further. 

Furthermore, based on international leading studies, we set 65 years as the cutoff value to divide the cohort into a younger and older age group. However, it is questionable whether this threshold will remain representative in an aging society [28]. The study spans from 2006 to 2020, and treatment practices for glioblastoma may have progressed during this period. Changes in diagnostic techniques, surgical approaches, or adjuvant therapies could impact the relevance of historical data to current clinical practice, which has to be considered. 

Although our study determined a large cohort, it only represented the results of a single center. Prospective multicenter studies are recommended to confirm our findings and to assess further predictors of the outcome of elderly glioblastoma patients. 

## 5. Conclusions

Elderly patients with glioblastoma (≥65 years) benefit from GTR, methylated MGMT promoter status, and hypofractionated radiation. Molecular pathways should be targeted for affecting PFS in the future. This study encourages investigations on elderly glioblastoma patients with a special emphasis on molecular characteristics such as PPM1D values of the tumor and lesion size, as well as postoperative treatment. As the incidence of glioblastoma has a peak in patients aged 75–84 years while the population is getting older, we must urgently focus on elderly patients with glioblastoma to identify clear treatment modalities for this subgroup.

## Figures and Tables

**Table 1:** Patient and surgical characteristics summarized.

Characteristics	Complete Cohort	<65 Years	≥65 Years
Period of time	2006–2020		
Number of patients	1004	528	476
Age (years), mean ± SD	61.91 ± 13.38	53.42 ± 8.77	72.70 ± 5.34
Sex (n, %)			
Male	589 (58.3%)	322 (61.0%)	267 (56.1%)
Female	415 (41.6%)	206 (39.0%)	209 (43.9%)
Tumor localization (n, %)			
Frontal		192 (36.5%)	156 (32.8%)
Temporal		145 (27.6%)	128 (26.9%)
Parietal		81 (15.3%)	88 (18.5%)
Occipital		55 (10.5%)	56 (11.8%)
Midline/Infratentorial/Bi-hemispheric.		55 (10.5%)	48 (10.1%)
Clinical presentation (n, %)			
Seizure	306 (30.5%)	185 (35.0%)	121 (25.4%)
Molecular status (n, %)			
MGMT methylation		203 (38.4%)	215 (45.1%)
Missing		77 (14.6%)	81 (17.0%)
Ki-67 proliferation index		18.96 ± 14.16	16.04 ± 10.70
KPS at admission (%)		83.34 ± 13.52	78.84 ± 14.9
KPS at discharge (%)		78.7 ± 16.32	70.54 ± 20.49
EOR			
Gross Total resection (GTR)		267 (50.6%)	229 (48.1%)
Subtotal Resection (STR)		145 (27.5%)	91 (19.12%)
Biopsy		116 (22.0%)	156 (32.8%)
Radiation Total Dose (mean, Gy)		58.3 ± 6.3	51.6 ± 14.5
Progression free survival (PFS, months)		8.0	7.0
Overall survival (OS, months)		15.73 ± 14.93	13.86 ± 15.73

**Table 2:** Mean Overall Survival and Progression-Free Survival in subgroup analyses.

Characteristics	Overall Survival (Mean ± SD)		Progression Free Survival (Mean ± SD)	
	<65 Years	≥65 Years	<65 Years	≥65 Years
Sex				
Male	14.73 ± 17.97	10.87 ± 14.23	12.90 ± 15.76	10.51 ± 18.63
Female	16.77 ± 15.88	8.95 ± 13.13	11.94 ± 11.38	9.88 ± 13.24
EOR				
Gross Total resection	15.48 ± 17.12	13.44 ± 14.34	12.77 ± 14.89	12.24 ± 15.69
Subtotal Resection	11.31 ± 7.45	9.79 ± 14.08	11.5 ± 11.81	9.73 ± 12.91
Biopsy	7.51 ± 5.98	4.12 ± 4.57	12.01 ± 10.22	6.21 ± 7.83
Molecular status				
MGMT methylation	19.10 ± 23.13	10.81 ± 14.68	14.07 ± 14.44	15.38 ± 21.71
unmethylated	12.63 ± 9.69	9.10 ± 12.68	11.14 ± 13.50	6.70 ± 3.60

**Table 3:** Analyses between the two groups pointing out the main differences.

Differences in Different Age Groups	
	***p***
**Mann-Whitney U Test**	
KPS at admission	<0.001 *
KPS at discharge	<0.001 *
Charlson Comorbidity Index	0.116
Radiation Dose	<0.001 *
Ki-67 proliferation index	0.005 *
Progression Free Survival	0.252
Overall Survival	0.068
**Chi-Square Test**	
Sex	0.125
Localization (Frontal vs. Temporal vs. Parietal vs. Occipital	
vs. Midline/Infratentorial/Bi-hemispheric)	0.445
MGMT methylation	0.001 *
Extent of Resection	
GTR	0.4
STR	0.002 *
Biopsy	<0.001*

* and digits in bold illustrate significant *p*-value (≤0.05).

**Table 4:** Univariate analysis and multivariate ordinal regression regarding overall survival in glioblastoma patients < 65 years and ≥65 years.

	<65 Years			≥65 Years		
Parameter	*p*-value	OR	95% CI	*p*-value	OR	95% CI
Sex	0.625	0.89	0.55–1.44	0.243	0.14	0.01–3.73
Localization						
Frontal lobe	0.674	0.9	0.55–1.47	0.732	1.84	0.06–9.95
Temporal lobe	0.146	1.45	0.88–2.38	0.678	2.24	0.05–10.83
Parietal lobe	0.574	0.82	0.42–1.62	0.873	0.72	0.01–37.47
Occipital lobe	0.225	0.65	0.32–1.31	0.899	0.72	0.00–12.44
M/I/Bi-hemispheric	0.707	1.16	0.53–2.54	0.439	0.12	0.00–17.34
Molecular status						
MGMT methylation	**0.002**	10.29	0.76–22.17	**0.03**	15.68	1.41–42.38
Extent of resection						
GTR	**0.034**	1.55	1.04–2.33	**0.003**	1.72	2.00–2.48
Biopsy	**0.014**	0.55	0.34–0.89	**0.001**	0.43	0.29–0.64
STR	0.951	0.99	0.63–1.55	0.186	1.37	0.86–2.20
Radiation Dose	0.176	1.21	0.91–1.56	**0.008**	1.02	1.01–1.04
Multivariate Analysis—Ordinal Regression						
Parameter	*p*-value	aOR	5% CI	*p*-value	aOR	95% CI
MGMT methylation	**0.001**	7.17	6.52–45.93	**0.001**	1.87	1.28–2.71
Extent of resection						
GTR	**0.046**	3.3	0.83–5.39	**0.001**	2.30	1.52–3.48
Biopsy	**0.049**	0.07	0.01–0.92	**0.001**	0.43	0.28–0.65
STR	0.344	0.79	0.49–1.28	0.114	1.98	1.15–3.42
Radiation Dose	–	–	–	**0.006**	1.25	1.06–1.46
Complete cohort—Ordinal Regression						
	*p*-value		OR		95% CI	
Ki-67 index	0.155		0.21		0.12–1.31	
Age	**0.001**		0.68		0.57–0.82	

bold illustrates significant *p*-value (≤0.05).

**Table 5:** Univariate analysis regarding progression-free survival in glioblastoma patients < 65 years and ≥65 years.

	<65 Years			≥65 Years		
Parameter	*p*-value	OR	95% CI	*p*-value	OR	95% CI
Sex	0.625	0.89	0.54–1.44	0.628	0.18	0.00–185.67
Localization						
Frontal lobe	0.674	0.90	0.55–1.47	0.204	9.36	0.08–29.61
Temporal lobe	0.496	5.04	0.05–28.80	0.224	13.27	0.05–35.24
Parietal lobe	0.490	0.13	0.00–15.75	0.109	0.002	0.00–4.05
Occipital lobe	0.276	0.03	0.00–17.77	0.464	0.04	0.00–9.55
M/I/Bi-hemispheric	0.786	2.71	0.00–16.67	0.501	0.01	0.00–2.48
Molecular status						
MGMT methylation	0.346	1.29	0.76–2.17	**0.001**	7.86	1.39–35.81
Extent of resection						
GTR	0.534	4.21	0.05–28.60	0.456	2.58	0.01–6.76
Biopsy	0.546	0.08	0.002–2.62	0.536	0.02	0.00–4.54
STR	0.752	0.45	0.003–6.58	0.685	0.13	0.00–23.11
Radiation Dose	0.820	0.94	0.54–1.63	0.211	1.43	0.82–2.50
Complete cohort—Ordinal Regression						
	*p*-value		OR		95% CI	
Ki-67 index	0.136		0.77		0.75–5.33	
Age	0.731		0.73		0.12–4.40	

bold illustrates significant *p*-value (≤0.05).

## Data Availability

Data are contained within the article.
